# Takayasu Arteritis in the Pre-Pulseless Phase Presenting as Pyrexia of Unknown Origin

**DOI:** 10.7759/cureus.45855

**Published:** 2023-09-24

**Authors:** Sooraj Unnikrishnan, Vaibhav Ingle, Abhishek Singhai, Kawal Pandita, Mahendra Atlani

**Affiliations:** 1 Department of Medicine, All India Institute of Medical Sciences, Bhopal, Bhopal, IND; 2 Department of Hospital Administration, All India Institute of Medical Sciences, Bhopal, Bhopal, IND; 3 Department of Nephrology, All India Institute of Medical Sciences, Bhopal, Bhopal, IND

**Keywords:** ct aortography, aortitis, fever of unknown origin, puo, takayasu arteritis

## Abstract

Pyrexia of unknown origin (PUO) or fever of unknown origin (FUO) is clinically challenging for a treating physician; it is also a conundrum for the patient until a definitive diagnosis is made. Despite extensive investigations, many cases of PUO may remain undiagnosed for a long time. In a resource-limited country like India, due to the limited availability of various diagnostic tests, a great many fever cases are classified as PUO. Here, we present a case report of Takayasu arteritis in its pre-pulseless phase, presented as PUO. Takayasu arteritis presenting as PUO in the absence of a pulse deficit is uncommon and rarely reported. The patient’s fever responded to steroids with methotrexate. The patient didn’t develop any vascular complications during the follow-up.

## Introduction

Fever may be the dominant presentation of many diseases, ranging from infectious, inflammatory, autoimmune, neoplastic, and related to drugs. Finding the etiology of fever without any localizing signs or potential diagnostic clues is troublesome. Petersdorf and Beeson first defined PUO as a fever greater than or equal to 38.3 C (101°F) on several occasions, persisting for three weeks, and the cause is uncertain even after one week of in-hospital investigations. PUO can be classified as classical, nosocomial, neutropenic, and HIV-associated PUO [1]. More than 200 conditions can present as PUO. Among the PUO cases, about 51% may remain undiagnosed, and among the diagnosed cases, about 17%-35% are infectious causes, 24%-36% are inflammatory causes, 10%-20% are neoplastic causes, and 3%-15% have miscellaneous causes [2]. Takayasu arteritis (TA) (pulseless disease) is a large-vessel vasculitis predominantly affecting young females. It mainly involves the aorta and its branches. The first case of TA was presented by an ophthalmologist, Dr. Takayasu, based on characteristic fundoscopy changes. Diminished or absent pulses can be seen in 84% to 96% of TA patients; vascular bruits in 80% to 94%; hypertension in 33% to 83%; and retinopathy is seen in 37% of patients [3]. The 1990 ACR (American College of Rheumatology) classification criteria for TA classified a patient as having TA if at least three out of six criteria were met, and these criteria have a sensitivity of 90.5% and a specificity of 97.8%. ACR criteria include the age of onset <40 years, claudication of extremities, decreased brachial artery pulse, blood pressure difference greater than 10 mm Hg, vascular bruits over a subclavian artery or aorta, and arteriogram abnormality [4]. Here, we share a case of TA presenting as PUO.

## Case presentation

A male in his 20s presented to our hospital with a fever for six months, episodes of blood in stool for four months, and abdominal pain for one month. Fever was intermittent, at least one episode/day, with temperatures ranging from 100 to 101°F. There was a history of spotting blood in stool for four months, which was intermittent and occurred once every two weeks. There was a history of weight loss and loss of appetite for six months. He had visited multiple primary care hospitals for this fever, but no definitive diagnosis could be made. He had been using paracetamol 2 to 3 times daily for six months. He was also receiving empirical antibiotics in the last three months. He also complained of epigastric pain for one month, which increased his food intake. There was no history of chronic cough, chest pain, exposure to tuberculosis, or other localizing symptoms. He had a continuous fever on presentation. On examination, there was no pallor/ icterus/lymphadenopathy/rash. His pulse rate was 110/min with a regular rhythm and a body temperature of 101°F on admission. His blood pressure in his left arm was 106/76 mm Hg, in his right arm 120/80 mm Hg, and in his right leg 146/76 mm Hg and 152/82 mm Hg in his left leg. Thrills or noises were absent. There was mild tenderness in the epigastric region on abdominal examination, with no organomegaly. Cardiovascular and respiratory examinations were non-contributory.

**Figure 1:**
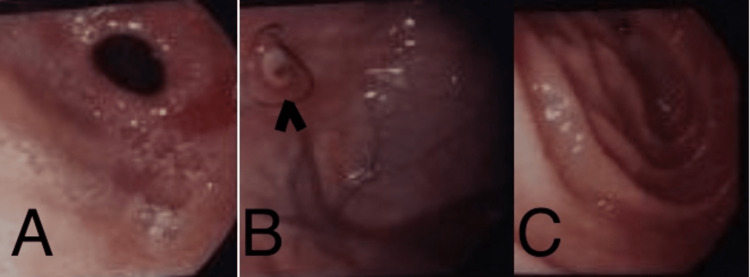
Upper gastrointestinal endoscopy A & B: Gastric fundus and body; gastric ulcer (black arrowhead) with mucosal hyperemia, C: Duodenum erosions are present

**Figure 2:**
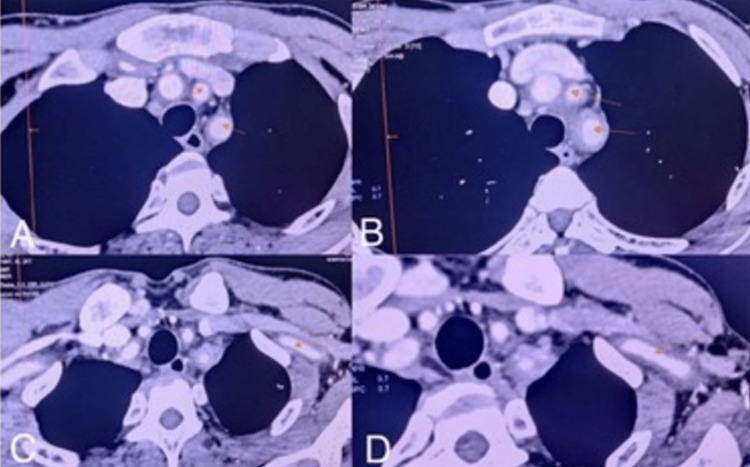
CECT thorax and abdomen show symmetrical luminal narrowing of the left subclavian artery, common carotid artery, and brachiocephalic trunk A & B: Left common carotid artery, and brachiocephalic trunk; C & D: Left subclavian artery (yellow arrows)

All routine laboratory investigations were normal except for the erythrocyte sedimentation rate (ESR) and C-reactive protein (CRP), which were elevated (Table 1). His chest X-ray was normal, and a contrast-enhanced CT scan (CECT) of the thorax and abdomen was planned. Meanwhile, he was investigated for epigastric pain, for which he had undergone upper GI endoscopy, which revealed gastric ulcers (two clean-based punched-out ulcers with an overlying slough present in the fundus) (Figure 1). Biopsies were taken from this ulcer, which was suggestive of acute or chronic gastritis, with no evidence of malignancy or Helicobacter pylori infection. Based on this finding, he was started on proton pump inhibitor (PPI) and syrup sucralfate, and his epigastric pain was relieved. A CECT scan of the chest and abdomen revealed mild circumferential symmetrical wall thickening involving the arch of the aorta, left common carotid, left subclavian, and brachiocephalic trunk (Figure 2). Subsequently, CT aortography was done, which also showed circumferential wall thickening involving ascending aorta (4.4mm, <50% narrowing), arch of aorta (3.7mm <50% narrowing), left common carotid (2.7mm, <50% narrowing), and left brachiocephalic trunk (2.5mm <50% narrowing). Short segments of moderate to severe narrowing of the first and second parts of the left subclavian artery (2.7mm, 50-60% narrowing) with severe narrowing of the third part of the left subclavian artery (3.6mm, 70-80% narrowing). These features were suggestive of aorto-arteritis, and based on CT aortography and elevated inflammatory markers, a diagnosis of TA was made. 

**Table 1:** Relevant investigations CRP: C-reactive protein; ESR: erythrocyte sedimentation rate; ALP: alkaline phosphatase; anti-HIV: anti-human immunodeficiency virus; USG W/A: ultrasound whole abdomen; UGI endoscopy: upper gastrointestinal endoscopy; Sputum AFB/CBNAAT: Acid fast bacilli/cartridge-based nucleic acid amplification test; COVID-19 RT-PCR: Coronavirus disease 19 reverse transcription-polymerase chain reaction test; VDRL: Venereal disease research laboratory; 2D ECHO: 2D (two-dimensional) echocardiography

Investigations	Day 1	Day 4	Day 10	Day 14
White blood cell count/μL	11290		9270	
Hemoglobin(gm/dL)	11.4		10.7	
Platelet count/μL	3,98,000		3,42,000	
Serum urea(mg/dL)	12			
Serum creatinine(mg/dL)	0.62			
CRP (mg/dL)	124 mg/dL		79.3	70.8
ESR (mm at the end of 1 hr)	100			68
Serum albumin (gm/dL)	3.32			
Serum ALP (IU/dL)	151			
Urine culture	Sterile			
Anti-HIV		Non-reactive		
USG W/A		Normal		
UGI endoscopy		Gastric ulcers		
Sputum AFB/CBNAAT	Not detected			
COVID 19 RT-PCR		Negative		
ANA (Immunofluorescence essay)		Negative		
Anti-HCV		Non-reactive		
HBsAg		Non-reactive		
T3, T4, TSH		Normal		
Sputum culture		No pathogenic organisms isolated		
Blood culture	Sterile	Sterile		
VDRL test			Non-Reactive	
2D ECHO		No vegetations		
Histopathology: Gastric ulcer biopsy			Acute on chronic gastritis	

Infectious aortitis, secondary to tuberculosis or syphilis, was an important differential. The patient’s history and the lack of stigmas and serologies associated with these infections made them less likely. Furthermore, a complete response to steroids and methotrexate and no flare of infection on subsequent follow-up for more than one year didn’t support the infectious cause.

The patient was started on prednisolone, methotrexate 15 mg subcutaneously weekly, tablet calcium, PPIs, and syrup sucralfate. The patient’s fever responded to medications and remained afebrile at subsequent visits. On follow-up for more than one year after the presentation, he did not develop any vascular complications like claudication/digital ischemia or renovascular hypertension. He underwent doppler ultrasound of the carotid arteries and subclavian arteries, which didn't reveal any stenosis.

## Discussion

TA is a rare idiopathic arteritis involving large vessels; it is relatively more common in Asia. Females in the 10-40 years age group are mostly affected by this disease. The pathogenesis of TA is not well understood, though T-cell-mediated cellular mechanisms have been proposed. The left proximal subclavian artery is usually involved first, and later, carotids, vertebral, and thoracic arteries may be involved. Characteristic clinical features include unequal radial pulses, subclavian or common carotid artery bruits, claudication, and secondary hypertension. Constitutional symptoms like fatigue, weight loss, and low-grade fever have been described in the early phase of TA. There are few case reports of TA presenting as PUO. On extensive searching of the literature, a case report from India of a 32-year-old female was found; she presented with recurrent fever, chest pain, and pleural effusion. She was treated with anti-tubercular therapy but showed no improvement. An invasive angiography revealed the right subclavian artery and carotid artery obstruction. The patient was started on steroids and methotrexate, and within three months, she significantly improved her symptoms [5]. Uthman et al. searched various search engines for TA presenting as PUO from 1968 to 1997; only two cases were identified [6]. The 2021 American College of Rheumatology guidelines suggested a combination of non-glucocorticoid and glucocorticoid use in patients with active TA rather than glucocorticoids alone [7]. Methotrexate and azathioprine are used as initial non-glucocorticoid agents. There are no well-established imaging or laboratory markers for monitoring disease activity. The guidelines recommended the use of non-invasive imaging over invasive imaging for the diagnosis of TA. High oral glucocorticoids are preferred over pulse therapy in severe acute TA. Regularly scheduled non-invasive imaging for routine clinical assessment every 3 to 6 months is recommended [7]. Earlier ACR and recent 2022 ACR/European Alliance of Associations for Rheumatology (EULAR) classification criteria for TA have used arterial insufficiency clinically and luminal damage on imaging in an appropriate clinical background to classify TA. However, application of these criteria for TA diagnosis in the pre-pulseless stage may be difficult in the absence of a very high index of suspicion.

## Conclusions

Takayasu arteritis is rarely present in the pre-pulselessness phase as PUO. Although infections are common differentials for PUO in the tropics, chronic inflammatory disorders like vasculitis should also be kept in mind. Early diagnosis and treatment of TA are important to prevent vascular complications like renovascular hypertension, claudication, and digital ischemia.
